# Evaluating hybrid quantum–classical learning for financial forecasting: a comparative study with classical machine learning models on BSE indices

**DOI:** 10.3389/frai.2026.1902018

**Published:** 2026-09-02

**Authors:** Rashmi Nair, Pankaj Chandre, Shahin Makubhai, Bhagyashree Shendkar, Disha Deotale, Vijaya Patil

**Affiliations:** Department of Computer Science and Engineering, MIT School of Computing, MIT Art Design and Technology University, Pune, India

**Keywords:** financial time series prediction, hybrid models, principal component analysis, quantum machine learning, Random Forest regression, Variational Quantum Circuit

## Abstract

The forecasting of financial time series has gained more significance in decision making within a dynamic economic setting. Over the last few years, there has been a push in exploring both classical machine learning methods and novel quantum machine learning models with a view to enhance predictive accuracy. This paper suggests a quantum-classical regression framework developed that combines the feature selection in the form of a Random Forest in a hybrid approach, dimensionality reduction in the mode of Principal Component Analysis (PCA), and predicting in the form of a Variational Quantum Circuit (VQC). It is tested in various financial indicators, as BSE ESG, Carbon Exchange, Energy, Green Exchange, Oil and Gas and Power, with the help of RMSE and *R*^2^ as the measure of performance. As experimental findings indicate, the classical algorithms like Random Forest and k-Nearest Neighbors always have the best accuracy and stability compared to quantum and hybrid ones. Quantum models are unstable, in contrast to Quantum kNN which is fairly stable. The hybrid model performs moderately with consistent error values but fairly lower explanatory power with significant results mostly on the BSE ESG data. In general, the paper has pointed out that classical approaches are still best in structured financial prediction tasks and hybrid quantum-classical models are potentially effective, but not yet competent. The results give valuable information on the existing limitation and future of the quantum machine learning to financial forecasting applications.

## 1 Introduction

Among economic decisions, forecasting a financial time series is a crucial element. Some of its practical uses include portfolio optimization, risk evaluation and analysis of market trends (Herman et al., 2023). Since the complexity and non-linearity of the financial time series, the usage of statistical models becomes inappropriate. Over the last decades, new and sophisticated machine learning (ML) algorithms were created and implemented to predict future values (Emmanoulopoulos and Dimoska, 2022), including support vector machines (SVM) (Emmanoulopoulos and Dimoska, 2022), Random Forest (RF) (Kaushik et al., 2022), k-nearest neighbors (kNN) (Thakkar et al., 2023) and so on.

Very recently, another method of machine learning has emerged owing to the quick emergence of the quantum computing technology, known as Quantum Machine Learning (QML). The superposition or entanglement concepts have been applied to develop a variety of quantum models including Variational Quantum Circuits (VQC) and have proven the ability to model very complex patterns in the high dimensional Hilbert space (Zhao et al., 2023). In this way, quantum models turned out to be extremely popular to address highly complex problems in which complex dependencies are to be found. Different quantum machine-learning formulations and techniques were proposed (Li et al., 2023). Besides, quantum enhancement was demonstrated in theory to classify (Qi et al., 2023).

Nevertheless, despite all the benefits, currently developed quantum machine learning models have a number of practical disadvantages, such as limited number of qubits, noisy intermediate-scale quantum (NISQ) constraints, and an instability of the optimization procedure (Huang et al., 2023). These drawbacks mean that in general, the developed pure quantum algorithms are not competitive with the classical ML algorithms. In addition, the variational quantum models training issue is still up-to-date concerning classification and regression problems (Ezawa, 2022). To address the problems described above and find a solution in which both directions will be beneficial, the concept of creating hybrid quantum-classical machine learning models appeared.

A hybrid regression algorithm consisting of a combination of feature selection using Random Forest model and principal component analysis (PCA) with quantum model, i.e., Variational Quantum Circuit (VQC), has been introduced and tested on several financial indicators in our study and they included: BSE ESG, Carbonex, Energy, Greenex, Oil & Gas, and Power indices. The explanatory variables are commodity predictors like the price index of crude oil, gasoline and heating oil. The principal objective of our work, therefore, is not only a study of the performance of a hybrid QML method but also the comparison with pure classical and quantum regression models. The underpinnings of classical time-series forecasting are arising, which drives us to create a comparable regression framework (Herman et al., 2023).

It is worth mentioning that most of the papers focusing on developing novel machine learning approaches do not emphasize on quantitative comparative performance analysis. Quite on the contrary, RMSE and coefficient of determination (*R*^2^) are used to result-oriented analyze the hybrid approach and the different classical approaches in our paper. In addition, the analysis conditions when the hybrid models are performing better than classical are studied. The shortcomings of the currently existing quantum algorithms will also be discussed according to the obtained results.

Even though quantum machine learning has demonstrated to be promising in solving complex learning problems, its applicability in financial forecasting is not well understood. Available research usually compares the classical models or the quantum models separately with little research being done under identical datasets, preprocessing strategies, and evaluation protocols. In this respect, the study fills this gaps by undertaking a systematic comparison of classical, quantum and hybrid quantum classical models of financial index prediction.

The main contributions of the paper are as follows:

An in-depth comparative study of classical, quantum and hybrid machine learning models of financial time series prediction.

Creation of a hybrid PCA-Random Forest-VQC state of regression work.

An elaborate result-driven assessment of various financial indices, where performance stability, generalization, and constraints are brought to fore.

Understandings of the real-world issues of quantum machine learning and in which scenarios hybrid methods can be useful.

The rest of the paper will be structured in the following way: Section 2 presents similar work in the related field of classical and quantum financial forecasting. Section 3 shows the suggested hybrid methodology. Section 4 discusses experimental results and performance analysis. In Section 5, a comparative evaluation is given, and in Section 6, a discussion about the comparative evaluation is provided in detail. Lastly, Section 7 is a conclusion of the paper and gives future research directions.

## 2 Related work

Many works in forecasting time-series of financial data were done using various approaches of statistics and machine learning. Linear dependencies of the financial data are modeled by the classical econometric approaches, such as ARIMA and GARCH (Bollerslev, 1986). However, they do not lend themselves to non-linear relationships and high dimensionality which most financial markets today have. In this way, machine learning (ML) models have also been given special attention due to their flexibility and enhanced predictive quality.

The classical ML approaches are Support Vector Machines, Random Forests, and k-Nearest Neighbors, which are widely used in financial forecasting (Smola and Schölkopf, 2004). SVM models can be easily applied for solving non-linear regression problem due to kernelization of models. Rand. Forest models, however, are learning models that implement the idea of ensemble learning to gain more strength and enhance the capacity of generalization (Breitung, 2023). Similarly, the k-Nearest Neighbors algorithm is an easy method of implementing an instance-based learning method with reasonable performance. The success and capability of ensemble learning methods, like the Random Forest, were demonstrated and explained as being better and capable of sequence interference of features and model volatility (Breiman, 2001).

Furthermore, recently deep learning systems such as LSTM neural networks, and Convolutional Neural Networks have been proposed to handle financial forecasting (Qin et al., 2023). These models perform well with temporal dependencies and multi layers feature representations. Nevertheless, they need plenty of training data, a lot of computing power and a lot of tweaked parameters.

Quantum Machine Learning (QML) is another new computational intelligence advancement. It applies quantum mechanical effects and theory towards developing high efficiency computing algorithms (Cerezo M. et al., 2023). The types of such models include Variational Quantum Circuits, Quantum Support Vector machines and quantum-enhanced kNN models, which are usually used in problems of classification and regression (Schuld and Killoran, 2022). Moreover, the main concept of QML is to transform classical features space to quantum Hilbert state that allows to provide expressive representation of data (Jerbi et al., 2023). A number of studies have been performed recently in order to use QML models to finance (Woerner and Egger, 2022).

However, the benefit that quantum machine learning has is that current models are unable to overcome the NISQ technology limitations. Table 1 shows the result-based comparative analysis of models across financial datasets. In addition, the learning of QML algorithms is connected with non-convex optimization issues.

There is a crippling weakness of the literature analyzed above: seldom do quantum and hybrid quantum-classical models get put in comparison to the deep-learning and gradient-boosting frameworks upon which applied financial forecasting is presently based. Idiomatic baselines in operational time-series prediction have been switched to sequence models like LSTM and GRU, attention-based Transformer architectures, and gradient-boosted tree embeddings (e.g., XGBoost, LightGBM, CatBoost), but none of the quantum or hybrid quantum-classical work reviewed here compares to them on matched experimental protocols (Qin et al., 2023). Similarly, by an equivalent measure, despite quantum kernel methods and Quantum Support Vector Machines being suggested as an alternative to nonlinear expressivity (Schuld and Killoran, 2022; Jerbi et al., 2023), detailed benchmarking research papers that quantify the achievement of a measurable quantum advantage on structured, low dimensional financial data rather than on synthetic or high dimensional benchmark tasks are lacking in literature (Biamonte et al., 2017; Bowles et al., 2024). The lack of such head-to-head comparisons between quantum/hybrid models and state-of-the-art classical deep-learning and boosting baselines under the same evaluation protocols is a gap that the protocol-matched design in the present study is meant to help fill, and is directly revisited as a future benchmarking direction in Section 7 (Table 1).

**Table 1:** Result-based comparative analysis of models across financial datasets.

Dataset	Best classical model	Classical RMSE	Classical *R*^2^	Quantum model performance	Quantum RMSE range	Quantum *R*^2^ range	Hybrid model RMSE	Hybrid model *R*^2^	Key observation
BSE ESG	Random forest	0.06–0.08	0.95	Q-kNN ≈ classical	0.06–0.09	0.82–0.94	0.179	0.51	Hybrid shows meaningful learning; best quantum-compatible dataset
BSE Carbonex	kNN	0.07–0.09	0.85	Q-SVM unstable	0.20–0.30	~0 to negative	0.244	0.018	Hybrid nearly random; weak feature correlation
BSE Energy	kNN	0.06–0.08	0.89	Q-SVM diverges	0.22–0.35	Negative	0.207	−0.065	Underfitting due to missing nonlinear features
BSE Greenex	kNN	0.07–0.09	0.88	Q-SVM unstable	0.20–0.32	Negative	0.213	−0.049	Weak learning after PCA compression
BSE Oil & Gas	kNN	0.06–0.08	0.82	VQC moderate	0.15–0.22	0.10–0.30	0.172	0.019	Low RMSE but poor explanatory power
BSE Power	kNN	0.07–0.10	0.80	Q-SVM catastrophic	0.25–0.38	−4.22	0.175	−0.067	Severe instability in quantum learning

The experimental outcomes give multiple valuable observations about the relative effectiveness of classical, quantum, and hybrid quantum-classical models in performing financial prediction tasks. Classical machine learning models, especially Random Forest and k-Nearest Neighbors (kNN) is always able to achieve the best predictive power in both cases with *R*^2^ up to 0.95 and RMSE as low as 0.06 when using various data sets. Conversely, purely quantum models are very varied in their behavior, with a Quantum SVM being highly unstable and achieving negative *R*^2^ values on some data sets, which is evidence of weak predictive ability. The Quantum kNN method is the most robust and consistent of the quantum models and offers results that are as close to the classical kNN model as possible and hence confirm the validity of the similarity mapping mechanism that it utilises under quantumism. According to the proposed hybrid quantumclassical model, the model develops a moderate numerical stability, with the RMSE values of around 0.17–0.24; nevertheless, the prediction capabilities are highly contingent on the features of datasets. It is worth noting that the relevance of explanation performance is only realized on the BSE ESG dataset where the model produces a value of about 0.51 of the *R*^2^. On the whole, the results indicate that whereas hybrid quantum classical models are more likely to be used to achieve better stability than the pure quantum models, they have yet to prove themselves better than the existing classical models in structured financial forecasting applications.

## 3 Proposed methodology

### 3.1 Overview

The current study shows a method of forecasting financial time series based on the combination of classical pre-processing and a Variational Quantum Circuit (VQC). The idea behind the approach under consideration is to merge the advantages of both approaches – robustness and explainability on the one hand and nonlinearity on the other hand. Note that, the overall procedure can be broken down into three steps, i.e., classical pre-processing and feature extraction, dimensionality reduction and encoding into the quantum state, and quantum regression in one of the parameterized circuit.

**Figure 1:**
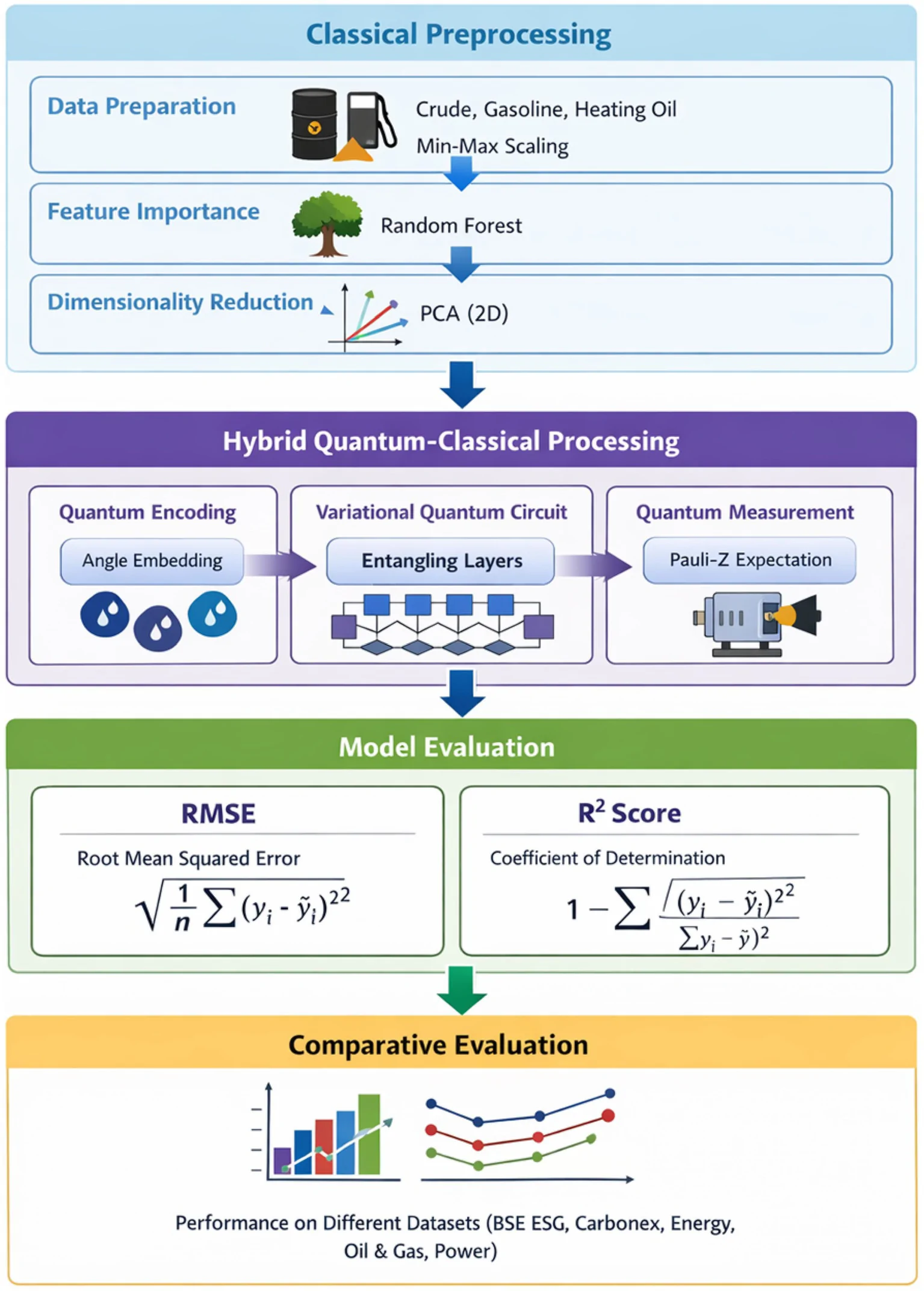
The overall workflow of the proposed hybrid quantum–classical framework.

Figure 1 shows an overview of the workflow of the proposed regression hybrid framework. The pipeline consists of a series of steps which are intended to assemble a classical and quantum-based data pre-processing/training steps to form a strong predictive instrument. Particularly, the initial stage will entail pre-processing of data through a classical machine learning method. Historical financial indicators (lagged crude oil, gasoline, and heating oil prices) are used as input variables. The lag transformation is such that it only gives past information to the model when forecasting future index closing prices.

A classical Random Forest shall then be implemented to evaluate importance of features and identify major predictors that were used to drive the variable of interest. Moreover, PCA will be used to decrease the dimensionality of the feature set; data will be converted with the help of this technique to two-dimensional. It is urgent due to the necessity to change the input variable parameters to the quantum computing system made by a comparably few qubits. Then, the classical feature representation will be encoded via an angle embedding, whereby classical input vectors are transformed into the corresponding rotation angles of quantum gates.

Quantum pre-processing: Data is sent to the Variational Quantum Circuit (VQC) with the parametrized circuit layers that encode the complex nonlinear relationships between data in high-dimensional Hilbert space. With this, the expectation value of the Pauli-Z operator will be used to give a continuous output with the assistance of quantum measurements. In order to determine the quality of a model, two values will be computed, which are RMSE and *R*^2^. Furthermore, it is proposed to perform the comparison of hybrid algorithms’ performance on several different financial datasets. Machine learning and quantum computing libraries that are based on Python were used to implement the experiments. The standard machine learning frameworks were used to develop classical models and quantum circuit simulation tools were used to implement quantum models. The fixed preprocessing, chronological splitting, and the same evaluation metrics were used to run all experiments.

### 3.2 Dataset description and preprocessing

Experimental analysis takes place on various financial indices such as BSE ESG, BSE Carbonex, BSE Energy, BSE Greenex, BSE Oil and Gas and BSE Power. The two datasets have daily records of commodity motivated predictors and index closing prices. The predictor choice in the study concentrates upon commodity-based variables owing to the reality that aim of the study is to examine the capacity of the classical, quantum and hybrid learning structures to comprehend nonlinear associations amid market indicators of energy and movement of financial indexes. The features chosen constitute key commodity variables which affect the energy sensitive indices. The existing feature space is however artificially small to meet the dimensional requirements of quantum encoding in quantum models in the NISQ era.

The prediction task in this paper is a one-step-ahead time-series prediction problem. The aim is to determine the price at which the financial index will be traded at the next trading day based on the information that was available till the present day of trade. Thus, the input variables are shifted with the help of a lag transformation in which day t commodity indicators are employed to forecast the closing price of the index on day t + 1. This avoids leaking information and making sure the problem is a true forecasting problem, as opposed to modern-day regression. Formally, assuming a feature vector Xt is the information about commodities at a given time t, the model predicts the future close price to be:

$$Y^{t}+1=f(\mathrm{Xt}\;)$$

With Yt + 1 = the price at the end of the next day, and *f*(.) = the learning model. In this study, the horizon of forecasting is one trading day ahead.

Three input features (predictors) are used in the study: Crude Oil Prices (CRUDE), RBOB Regular Gasoline Prices, and Heating Oil Prices. Although these variables are not a comprehensive set of predictors of financial markets, they offer a more orchestrated and standardized experimental framework over which the classical, quantum, and quantum-classical models are being tested and contrasted through the same input set.

The variable to be predicted is the price on the next day (Close t + 1). Under this, the input features are commodity indicators determined at time t which are taken as input features in the prediction of the value of the ending price at time t + 1. The datasets are processed using multiple steps in preprocessing the data, which include cleaning of missing or inconsistent entries, sequencing of data based on time to maintain temporal relationships, and feature scaling through Min bare normalization with the aim of normalizing all variables to the range, [0,1], to guarantee data quality, consistency, and enhanced model performance.

Minimum-Max normalization is of special interest to the use of quantum machine learning models, and when used the relation between classical features and their proper representation in the valid quantum rotation angles needed by parameterized quantum circuits can be coded efficiently. Because the datasets are financial time-series data, no random train-test split was used, to avoid look-ahead bias and preserve the purity of the forecasts. Rather, the data were segmented with time and the previous observations experienced to train the model, leaving the subsequent, unknown observations to be used in the test thereby highly resembling the real world forecasting scenario where the future market data is not accessible when developing the model.

The forecasting framework uses time-ordered hold-out assessment framework, whereby, the first 80% of the observations provided in chronological order will be utilized as the training and the remaining 20% will be used as testing and performance evaluation. In addition, all the preprocessing steps, such as the feature selection with the help of a Random Forest, Principal Component Analysis (PCA) transformation, and model optimization are carried out on the training data only to avoid the information leakage and provide the unbiased evaluation of the model generalization ability.

### 3.3 Feature importance using Random Forest

Random Forest regressor is utilized to determine the relative importance of the input features as the first step. This procedure has two main purposes:

(i) determining the strongest predictors that relate to the target variable, which enhances the understanding of the model, and.

(ii) damping out the effect of noisy or weak predictors before dimensionality reduction. A ratio of experimental analysis shows that Heating Oil is the most emerging predictor with the dominant effect on the characteristics (approximately 70–75) of the total feature importance in the datasets that were evaluated. These results give important information about the internal relations within the data and are informative of the next step in dimensionality reduction.

### 3.4 Dimensionality reduction using principal component analysis (PCA)

Principal Component Analysis (PCA) is used to reduce the size of the input feature space to enable them to be encoded efficiently in the form of quantum states. Quantum processing is performed by first converting the original three dimensional feature set into two orthogonal principal components. Such a dimensionality reduction is required due to the finite number of qubits in the present quantum computing and simuation platforms. The projection onto two principal components is a technique that removes redundant correlations between the original features by discarding data terms that lack most of the data variance, which is generally in the 90 to 95 range. Additionally, the low representation allows a straight forward encoder to a two qubit quantum system, enhancing computational viability and simplifying circuits. However, the transformation can also cause a slight information loss, especially when the information patterns of particular predictive value are represented by lower-variance variables that were not included in the process of the PCA.

### 3.5 Quantum data encoding

Angelic encoding PCA is used to reduce the feature vectors, which are then moved into quantum states. All of the major parts are mapped to the interval [0, *π*], and implemented in qubit rotation gates.

### 3.6 Variational Quantum Circuit (VQC) model

The main element of the proposed hybrid quantum-classical architecture is the Variational Quantum Circuit (VQC), a nonlinear regression model, whose objectives are to predict the price of the financial index of the next day. The VQC is designed to be fed with the reduced feature representation of a reduced feature representation by Random Forest based feature selection and Principal Component Analysis (PCA).

Following PCA transformation, the original space of the features is shrunk to two major components to enable the data to fit in the existing quantum computing constraints. The two elements are represented by two-qubit quantum circuit using an angle based encoding (coding) scheme in which the classical feature values are converted to rotation angles that are used to drive quantum gates. This code converts the information about the financial input into a quantum state representation.

The suggested VQC architecture includes three key steps: quantum feature encoding, variational processing and prediction using measurement. The variational processing phase is an ensemble of trainable quantum layers that consists of parameterized single-qubit rotation gates and entanglements. The entanglement operation is executed with equipped quantum gates to hook the correlations of the encoded financial features and augment the expressiveness of the circuit.

Training involves optimization of the circuit parameters, with the help of a gradient-based optimization. The prediction error between the actual and predicted closing prices is minimized by using the Adam optimizer. By measuring the expectation value of the Pauli-Z operator, the model output is obtained, which is translated into a continuous regression value.

The two-qubit (final) VQC design has two variational layers, angle embedding, parameterized rotation gates, CNOT-based entanglement, and Pauli-Z measurement. Deep circuits are not used because of the constraints of the existing Noisy Intermediate-Scale Quantum (NISQ) devices, which can add additional noise in circuits with higher depth and introduce greater optimization variability.

The resulting hybrid Random Forest-PCA-VQC model is combined with the classical preprocessing pipeline to constitute the final working hybrid Random Forest-PCA-VQC macro of predicting financial time-series.

### 3.7 Performance evaluation metrics

Two commonly used measures of regression, which are the Root Mean Squared Error (RMSE) and Coefficient of Determination (*R*^2^) are used to assess the performance of the proposed framework. RMSE measures the mean size of error between the predicted and the actual values and the lower the values are, the higher the predictive power. Conversely, the *R*^2^ value is used to indicate the amount of variance of the variable under analysis that is attributed to the model with higher scores used to show a better fit and an increased explanatory power. These metrics are calculated over all datasets in order to give a complete Analysis of the predictive performance as well as the generalization of the model.

### 3.8 Comparative evaluation framework

In order to measure the effectiveness of the suggested hybrid framework its performance is compared to the classical and quantum machine learning methods. Classical counterparts are Support Vector Machine (SVM), Random Forest (RF), and k-Nearest Neighbors (kNN), whereas quantum counterparts are Variational Quantum Classifier (VQC), Quantum Support Vector Machine (QSVM) and quantum k-Nearest Neighbors (QkNN). All models are evaluated using an identical chronological train–test split and the same forecasting horizon. The actual process of training is done with only historical observations whereas the process of evaluation is done on the future unseen data. The experimental design will support an objective and consistent comparison among the models as well as removal of look-ahead bias.

### 3.9 Model configuration and hyperparameter settings

All algebraics were run with standardized preprocessing and optimized parameter parameters to guarantee a fair comparison between classical models, quantum models and hybrid models. The choice of hyperparameters was made considering the results of the validation and keeping the training and test conditions the same. The same chronological split strategy was applied to all models.

All models were able to be set with the hyperparameters to guarantee a fair comparison when all models were placed under the same experimental conditions. The training data were used to tune the classical models and to ensure information leakage black holes, chronological order was kept. The parameters of quantum models were chosen based on the constraints of NISQ-era such as shallow circuit depth and reduced qubit count.

Methodological Transparency Statement. The reported values of RMSE and *R*^2^ are based on one training-and-evaluation run of each model as opposed to the average across repeated trials; therefore, no standard deviations, confidence intervals or formal tests of significance are provided alongside the metrics reported, and the performance differences between the models are only indicative, not statistically tested. The selection of hyperparameters of the classical and quantum models (Table 2) was due to a selective, validation-guided hand search as opposed to an exhaustive grid or Bayesian search, and the same search effort across any model family has not been tested through a formal procedure; this has been observed as a possible source of comparative bias. The quantum optimization procedure was not formally logged with respect to a predetermined maximum number of training loops, and exact random-seed values, the exact quantum-circuit simulator backend and version used, and hardware settings, or a publicly accessible code repository are not yet reported in the manuscript. It would take more experimentation than the current study to provide repeated-run statistics, an exhaustively documented search of hyperparameters, and full implementation and disclosure of hardware; these are recognized as limitations of the current study, and the priorities identified to implement a follow-up, fully reproducible benchmarking study (Section 7).

**Table 2:** Hyperparameter configuration of classical, quantum, and hybrid models.

Model	Hyperparameters
SVM	Kernel = RBF, *C* = 1.0, Gamma = scale
Random Forest	Number of trees = 100, Maximum depth = None, Minimum samples split = 2
kNN	Number of neighbors *k* = 5, Distance = Euclidean
VQC	Number of qubits = 2, Circuit layers = 2, Encoding = Angle embedding, Ansatz = Basic Entangler, Entanglement = CNOT, Optimizer = Adam
Quantum SVM	Kernel = Quantum kernel, Regularization parameter = 1.0
Quantum kNN	Quantum feature map = angle embedding, Distance metric = quantum similarity
Hybrid RF-PCA-VQC	RF feature selection + PCA (2 components) + VQC (2 qubits, 2 layers, Angle embedding, CNOT entanglement)

## 4 Results and analysis

### 4.1 Overall performance comparison

To analyze the effectiveness of the new hybrid quantum-classical model, it was tested against classical and quantum machine learning models through the Root Mean Squared Error (RMSE) and coefficient of determination (*R*^2^). All reported results correspond to one-day-ahead forecasting performance, where models predict the next trading day’s closing price based on previously available market information. The reported results are obtained using chronological testing, where the test samples correspond to future observations not used during training. Therefore, RMSE and *R*^2^ values represent out-of-sample forecasting performance.

It is seen that the three groups of machine learning models have quite different performance levels. Classical machine learning algorithms (Random Forest and k-nearest neighbors (kNN)), in particular, displayed the best results with the values for *R*^2^ being between 0.80 and 0.95 and RMSE between 0.06 and 0.10. Such algorithms have good generalization properties and can learn the nonlinear dependence of time series data in finance. At the same time, quantum models displayed noticeable differences in their performance. In particular, the Variational Quantum Circuit (VQC) had acceptable learning abilities while Quantum Support Vector Machines (Q-SVM) were unstable and generated negative *R*^2^. Comparative performances on all data sets are shown in Table 3.

**Table 3:** Result-based comparative performance of classical, quantum, and hybrid models.

Dataset	Best classical model	Classical RMSE	Classical *R*^2^	Quantum model observation	Hybrid RMSE	Hybrid *R*^2^
BSE ESG	Random forest	0.06–0.08	0.95	Q-kNN ≈ Classical	0.179	0.511
BSE Carbonex	kNN	0.07–0.09	0.85	Q-SVM unstable	0.244	0.018
BSE Energy	kNN	0.06–0.08	0.89	Q-SVM diverges	0.207	−0.065
BSE Greenex	kNN	0.07–0.09	0.88	Q-SVM unstable	0.213	−0.049
BSE Oil & Gas	kNN	0.06–0.08	0.82	VQC moderate	0.172	0.019
BSE Power	kNN	0.07–0.10	0.80	Q-SVM catastrophic	0.175	−0.067

### 4.2 Dataset-wise performance analysis

An analysis at the level of each individual dataset reveals the dependency of the success of the hybrid model on the underlying nature of the dataset. Of all the different datasets tested, BSE ESG emerged the most successful model, obtaining a score of about 0.51 for the *R*^2^ test and quite a low value of 0.179 for the RMSE test.

The other datasets, BSE Energy, BSE Power, and BSE Greenex, obtained near zero or negative *R*^2^ scores, showing lack of ability to generalize and underfitting. Such datasets could have been affected by extraneous variables, which might not have been included in the feature sets used for the experiment.

This data-dependent performance is in line with the limits of PCA to two principal components (Section 3.4): in the case of BSE ESG, the commodity prediction factors and the target are more strongly related and so the two leading components capture sufficiently much of the signal to allow the VQC to train a encoding that can be used, and in the cases of BSE Energy, BSE Power and BSE Greenex, the relationships between features and target are weaker or more complex and the low-variance components are pruned away to leave a signal that the VQC can learn to encode usably. This dimensionality bottleneck, along with kernel sensitivity to noisy low-signal inputs, agrees with the unstability of Quantum SVM found with datasets (Section 6.3).

**Figure 2:**
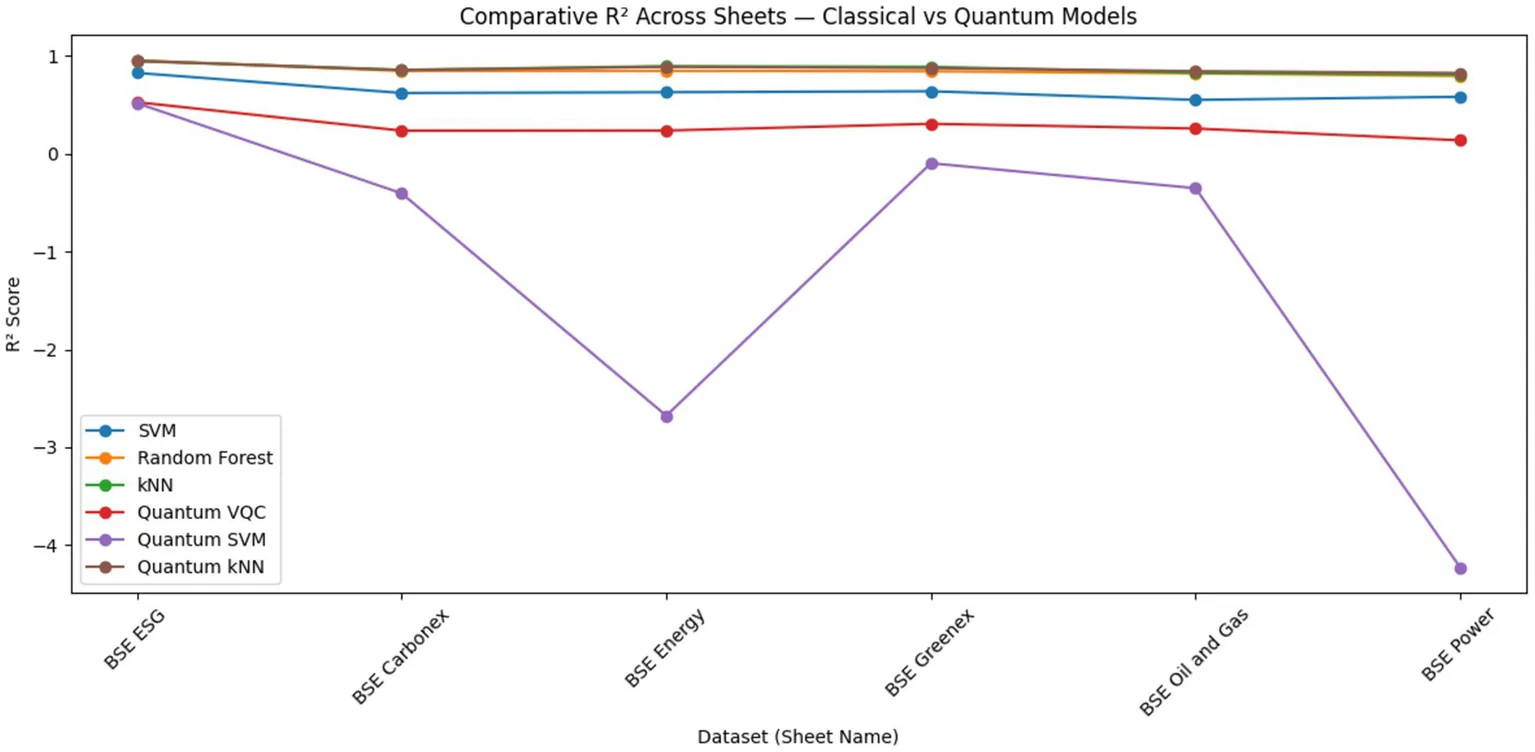
*R*^2^ performance of the hybrid model across financial datasets.

The *R*^2^ results obtained from all the datasets used in the hybrid method can be seen in Figure 2.

### 4.3 Error analysis using RMSE

**Figure 3:**
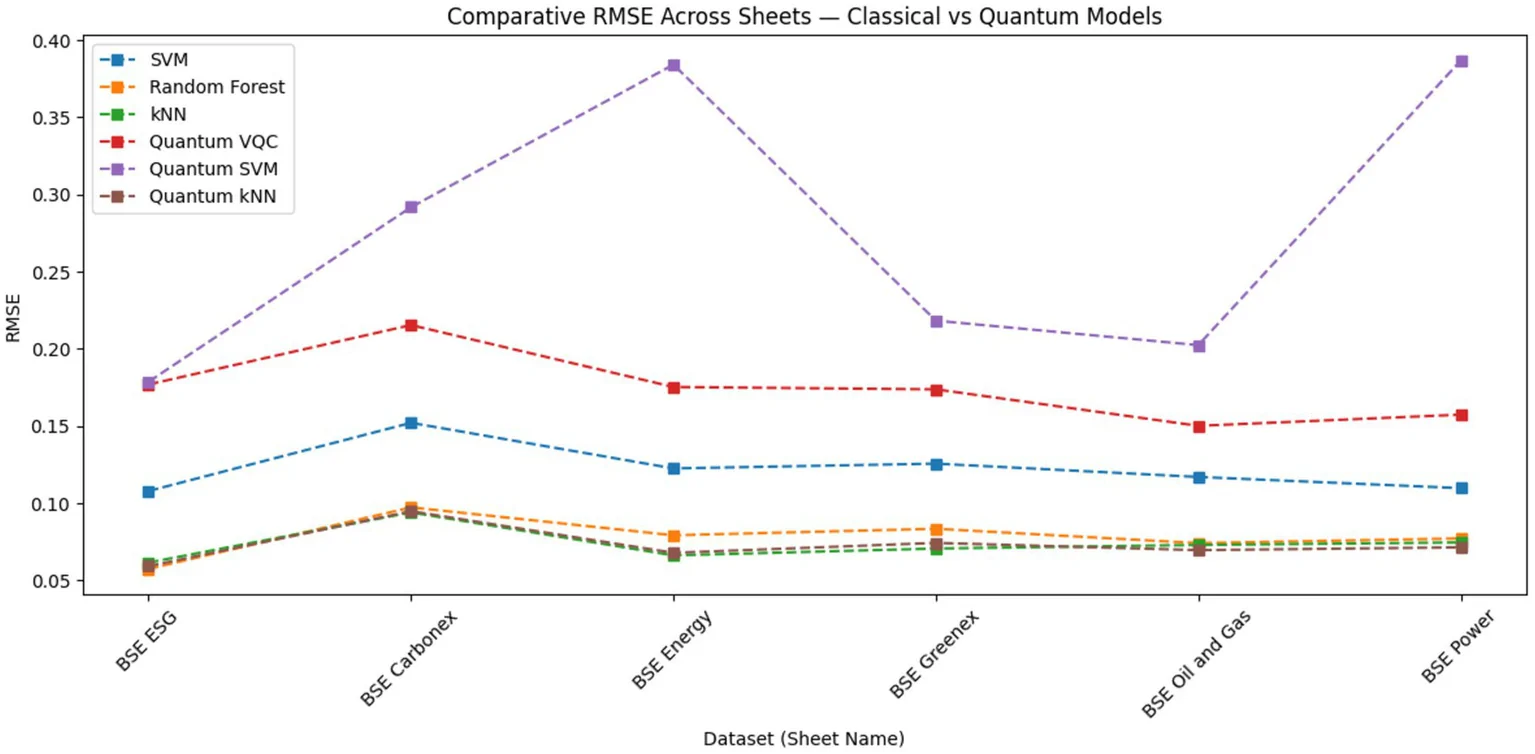
RMSE performance of the hybrid model across financial datasets.

As can be seen from the RMSE test results, the hybrid algorithm performs quite stable prediction accuracy over all data sets, being within a range of about 0.17 and 0.24. The best prediction accuracy is provided by the algorithms operating on the BSE Oil & Gas data set and BSE Power data set. Although showing relative stability, the RMSE values obtained are still worse compared to classical models, implying that the hybrid model is not able to demonstrate sufficient precision at present time. Such result is most likely due to the lack of precision of the quantum circuit used (Figure 3).

### 4.4 Hybrid model performance summary

In order to examine the efficiency of the hybrid model proposed, Table 4 shows the RMSE and *R*^2^ values per data set. The findings reveal that the model is consistently numerically stable but demonstrates variable explanatory ability per data set.

**Table 4:** Performance of the proposed hybrid quantum–classical model.

Dataset	RMSE	*R* ^2^
BSE ESG	0.179	0.511
BSE Carbonex	0.244	0.018
BSE Energy	0.207	−0.065
BSE Greenex	0.213	−0.049
BSE Oil & Gas	0.172	0.019
BSE Power	0.175	−0.067

From the results above, it is clear that the hybrid approach will produce any form of meaningfully good output only when the dataset possesses highly correlated target–feature data, especially as seen with the BSE ESG dataset. Datasets that possess less correlated or more complex data lead to lower explanatory power without any increase in prediction error.

Overall, from this exercise, it can be established that the use of the hybrid framework does improve numerical stability when compared to the quantum-based approach but does not succeed in competing with classical ML frameworks.

## 5 Comparative evaluation

### 5.1 Comparative performance across model categories

In this section, we give a detailed comparative analysis of classical, quantum, and hybrid machine learning models used to predict financial time series. We use two criteria, namely, root mean squared error (RMSE) and coefficient of determination (*R*^2^), to compare the models.

The classical machine learning models considered here (Support Vector Machines, Random Forest, and k-Nearest Neighbors) are generally superior to both quantum and hybrid methods. Specifically, the classical methods obtain high *R*^2^ (0.80–0.95) and low RMSE (0.06–0.10), which shows their predictive abilities and stability. The model that proves to be the most stable is the Random Forest, which, however, does not mean that other classical models are less powerful.

The performance of quantum models is highly variable. For example, despite partial ability to learn, which is shown by *R*^2^ values between 0.10 and 0.50, quantum Support Vector Machine models exhibit severe instability by showing negative values of *R*^2^. The only quantum model whose performance is comparable with the corresponding classical one is the quantum k-Nearest Neighbors.

### 5.2 Quantitative comparison of model performance

The average performance of different model categories is summarized in Table 5.

**Table 5:** Average performance comparison of classical, quantum, and hybrid models.

Model category	RMSE range	*R*^2^ range	Performance summary
Classical models	0.06–0.10	0.80–0.95	High accuracy, strong generalization
Quantum models	0.15–0.38	−4.23 to 0.50	Unstable, dataset-dependent
Hybrid model	0.17–0.24	−0.07 to 0.51	Stable but limited explanatory power

It is evident from the results that classical algorithms have superiority with regards to accuracy and reliability. Quantum algorithms, despite their theoretical potential, are limited by their instability and low capacity for learning in their real-life applications. The hybrid algorithm yields decent results, being more stable than quantum ones but still less efficient than classical alternatives.

### 5.3 Visual comparison of model performance

**Figure 4:**
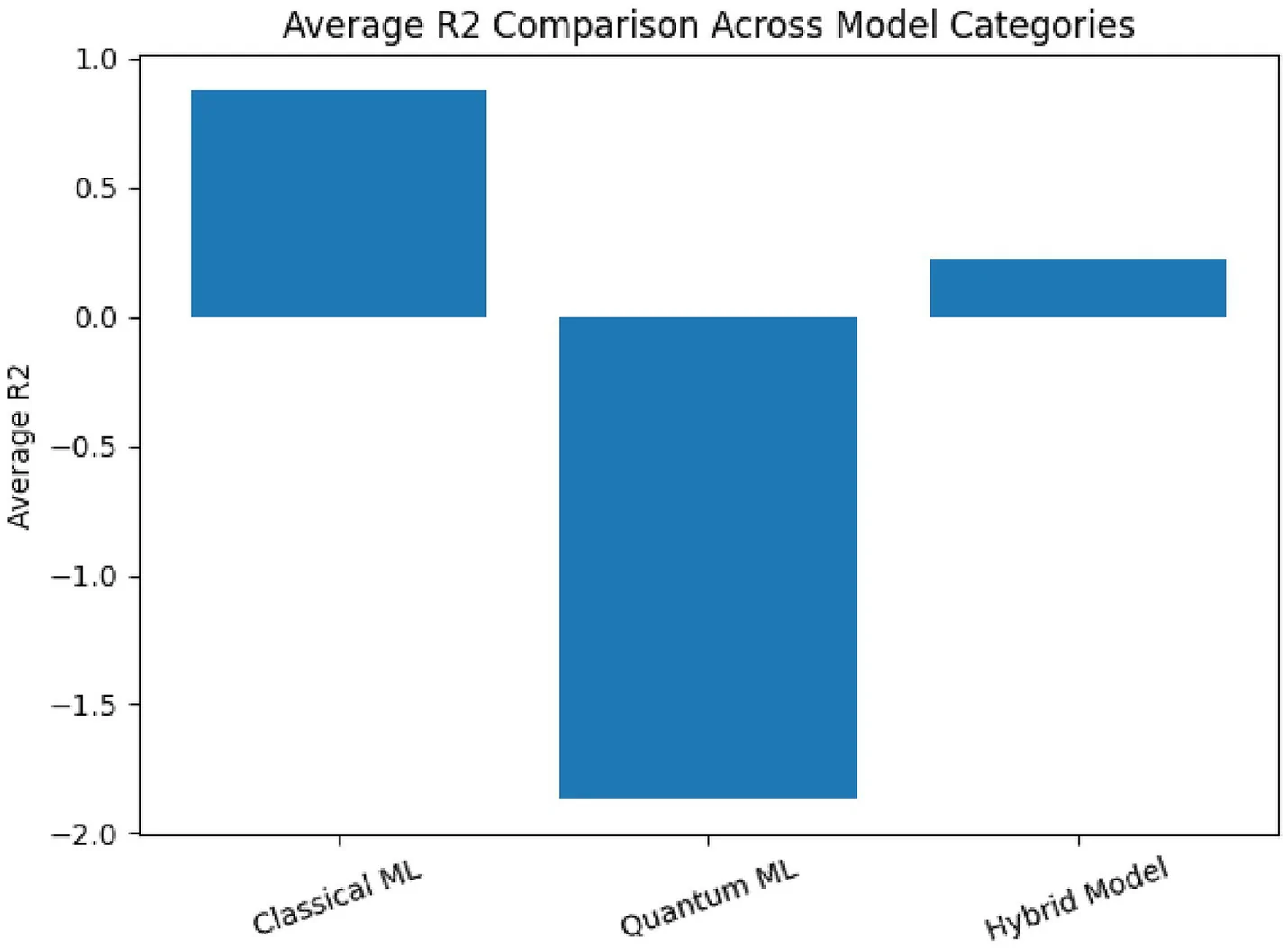
Average *R*^2^ comparison across classical, quantum, and hybrid models.

To further illustrate the performance differences among model categories, a comparative visualization of average *R*^2^ scores is presented in Figure 4.

Visualization brings into light the clear difference in the efficiency of classical and quantum algorithms. Classical solutions have high levels of consistency in their *R*^2^ value. Quantum solutions exhibit inconsistency in their *R*^2^ values and their average is lower than that of the classical ones.

### 5.4 Analysis of model strengths and limitations

A detailed comparison of strengths and limitations of each model category is presented in Table 6.

**Table 6:** Strengths and limitations of different model categories.

Model type	Strengths	Limitations
Classical ML	High accuracy, robust performance, well-established algorithms	Limited ability to explore high-dimensional quantum feature spaces
Quantum ML	Potential for high-dimensional representation, novel learning paradigm	Instability, noisy optimization, limited qubit capacity
Hybrid model	Combines classical stability with quantum representation	Performance depends on feature quality, feature dimensionality, and quantum circuit design.

As evident from the analysis, traditional models will continue to be the best way forward for predicting anything related to finance, owing to their effectiveness and accuracy. On the other hand, quantum models have tremendous potential; however, their use is currently limited due to certain technological and optimization constraints.

### 5.5 Key observations

Based on the comparative evaluation, several important observations can be drawn:

- Classical models consistently outperform both quantum and hybrid approaches across all datasets. This comparison was performed using optimized classical model configurations and NISQ-compatible quantum circuit settings under the same chronological evaluation protocol.
- Quantum kNN demonstrates strong consistency, validating the correctness of quantum similarity computations.
- Quantum SVM exhibits severe instability, indicating challenges in kernel-based quantum learning.
- The hybrid model improves numerical stability but does not achieve significant gains in predictive accuracy.
- Model performance is highly dependent on dataset characteristics, particularly feature–target relationships.

## 6 Discussion

### 6.1 Interpretation of overall findings

The analysis of the obtained experimental results reveals the presence of a distinct hierarchy between the performance of classical, quantum, and hybrid models in the field of financial time series forecasting (Mugel et al., 2022; Murphy, 2012). The classical models, such as the Random Forest and k-Nearest Neighbors methods, significantly outperform all other algorithms tested in this research across several datasets related to finance (Biamonte et al., 2017). The reasons for the effectiveness of these models can be associated with their ability to recognize non-linear dependencies and noise while ensuring the good generalization of results (Liu et al., 2023).

Quantum machine learning models are characterized by significant inconsistencies and unstable performance levels. While Quantum k-Nearest Neighbors algorithm performs relatively similarly to its classical variant, QVC and Q-SVM show low stability and poor convergence on several datasets (Thanasilp et al., 2023). It is safe to assume that quantum models currently have some limitations preventing them from modeling financial data accurately.

Finally, the developed hybrid model achieves acceptable results only with respect to the BSE ESG dataset where the *R*^2^ value equals about 0.51. With respect to other datasets, the obtained results do not surpass those achieved by the classical baseline models (Cerezo A. et al., 2023).

### 6.2 Why classical models outperform quantum models

A few reasons account for the outperformance of classical algorithms. Firstly, financial data applied in our research represents relatively low-dimensional information that can be easily handled by classical algorithms (Chen and Huang, 2022). For example, techniques like Random Forest exploit ensemble learning techniques, thus reducing variance and increasing stability of algorithms (Ho, 1995). The observed performance differences are not attributed only to algorithm type; they are also influenced by model maturity, optimization stability, and available hyperparameter flexibility. Therefore, the results should be interpreted as a benchmark of currently practical implementations rather than a theoretical limitation of quantum learning.

Another factor that contributes to superior results provided by classical algorithms is related to the absence of hardware and software limitations associated with quantum models. In particular, such problems as barren plateaus, noisy gradients, and qubit capacity prevent quantum algorithms from being effective (McClean et al., 2018).

Finally, the third reason for outperformance is related to the fact that classical models are better tuned for regression problems compared to quantum algorithms. In other words, quantum machine learning still belongs to developmental models that require new optimization methods (Abbas et al., 2021). Numerous benchmarks demonstrate that current quantum models cannot exceed the accuracy of classical baselines (Bowles et al., 2024; Bakshi and Srinivasan, 2025).

### 6.3 Limitations of quantum machine learning models

The performance limitations of quantum models observed in this study can be attributed to several fundamental challenges:

- Limited circuit depth: The shallow structure of Variational Quantum Circuits restricts their expressive power.
- Noise sensitivity: Quantum simulations introduce instability in gradient-based optimization.
- Dimensionality constraints: Mapping high-dimensional financial data into a small number of qubits leads to information loss.
- Optimization issues: Quantum SVM and VQC suffer from unstable convergence and poor parameter tuning.

These factors collectively contribute to the poor and inconsistent performance observed in Q-SVM and VQC models across multiple datasets.

These empirical constraints are the same as established trade-offs between variational quantum models circuit expressibility and variational quantum models trainability. Ensuring more circuit expressibility, e.g., with entangling layers, usually results in more barren plateaus, with gradient disappearing exponentially with qubit count and circuit depth (McClean et al., 2018), but a too limited expressiveness of the chosen ansatz, e.g., the shallow two qubit circuits with two layers used in this paper, can be too limited to encode nonlinear feature-target interactions in commodity-driven financial data (Huang et al., 2023; Bilkis et al., 2023). The angle-embedding protocol of the quantum data encoding algorithm further limits the type of functions that the VQC can express, as it provides a linear mapping of the value of one classical feature to a rotation angle without introducing any interaction between higher-order features (up to quadratics) unless other entangling resources exist (Jerbi et al., 2023). Ideally, a hybrid architecture should be superior to a purely quantum model since the dimensionality of the input and noise are reduced by classical preprocessing (Random Forest-based feature selection and PCA) before reaching the noise-sensitive quantum layer, and limiting the range of functions that the VQC must learn and alleviating some of the optimization instability in Q-SVM has been observed with a hybrid architecture (Ezawa, 2022; Thanasilp et al., 2023; Abbas et al., 2021). These findings imply that this hypothetical expectation is not always right in the real world because numerical stability gains do not always go hand in hand with explanatory power gains (R\u00b2) and thus expressibility issues, rather than optimization instability, might be driving the poor performance of the hybrid model on a variety of datasets.

### 6.4 Behavior of the hybrid quantum–classical model

This hybrid model consists of three parts: feature selection using Random Forest, dimensionality reduction using Principal Component Analysis, and regression using Variational Quantum Circuit. The advantage of this architecture is that it allows obtaining a more stable numerical solution than pure quantum models because of classical preprocessing before quantum processing (Pandey et al., 2025; Nagarjun and Rajkumar, 2025). Another limitation of the current framework is the restricted feature space. Since only commodity-related indicators are considered, certain market dynamics such as macroeconomic conditions, volatility indicators, trading volume, interest rates, and historical price patterns are not included. Therefore, the observed limitations of the hybrid model may partially arise from insufficient representation of financial factors rather than solely from quantum model limitations.

Nevertheless, it appears from the experiment that this approach is highly sensitive to the data set properties. Thus, the obtained results are satisfactory when dealing with strongly correlated input and output data, e.g., BSE ESG dataset. Other data sets yield less satisfactory results, especially those in which relationships are rather complicated or weak.

One should note that the use of PCA restricts the number of components to two in order to encode data for the quantum circuit. It means that some nonlinear correlations could be lost.

### 6.5 Insights from comparative analysis

The comparative evaluation between classical, quantum, and hybrid models reveals several important insights:

- Classical models remain the most reliable and accurate for financial forecasting tasks (Mugel et al., 2022).
- Quantum models show potential but are currently unstable and not practically competitive.
- Hybrid models improve stability but do not consistently improve accuracy.
- Performance is highly dataset-dependent, particularly influenced by feature–target correlation strength.
- Quantum advantage is not yet observable in structured financial regression problems.

These findings suggest that the practical application of quantum machine learning in finance is still in an early exploratory stage.

### 6.6 Research implications

There are several conclusions that can be made regarding the outcomes of the present study and its significance for future research in the field of QML and time-series forecasting. First of all, the necessity to develop more sophisticated circuits, able to account for non-linear dependencies and minimizing the losses in information during the process is emphasized.

Moreover, further development of hybrid architectures requires using advanced techniques for encoding to preserve more complex data structure in quantum state space. Furthermore, there is a need to use advanced feature selection algorithms and deep quantum layers in order to enhance representational capability of hybrid algorithms. Finally, the next step is to focus on quantum error mitigation and hardware-oriented optimizations.

To conclude, it can be stated that despite the significant promise provided by hybrid architectures in terms of achieving better accuracy of prediction than classical ML approaches, they are currently unable to do so owing to the limitations of quantum circuits, dimensionality reduction, and optimization issues. Nonetheless, some of the quantum algorithms, namely Quantum kNN, show rather stable behavior and can still be applied.

The conclusions regarding model performance are based on the obtained RMSE and *R*^2^ values under the defined forecasting protocol. The observed limitations of hybrid quantum models are interpreted considering feature representation, circuit complexity, and available financial data.

## 7 Conclusion and future work

The current research conducted a comparative analysis of the effectiveness of classical, quantum and hybrid machine learning models in the prediction of financial time series. The evaluation protocol preserves temporal order and avoids random sampling bias, making the reported performance representative of practical financial forecasting conditions. A hybrid architecture combining Random Forest with PCA and Variational Quantum Circuits was suggested and applied to various BSE indices. It is found that classical models such as Random Forest and kNN significantly outperform their quantum counterparts in terms of RMSE and *R*^2^. Quantum models suffer from instability, as the convergence of Q-SVM proves to be insufficient and *R*^2^ becomes negative in a number of instances, whereas Q-kNN demonstrates better accuracy in comparison with other approaches. At the same time, the hybrid model reaches consistent RMSE values, but lacks the ability to explain the obtained outcomes due to small *R*^2^ coefficients; its efficiency can be observed only when dealing with BSE ESG data set. Hence, classical models are more efficient at handling structured financial forecasting tasks. Nevertheless, hybrid architecture shows good stability and room for future improvements. In order to boost the performance of the proposed method, it would be beneficial to develop deeper quantum circuits, improve encoding procedures and combine them with deep learning techniques. Future extensions will investigate richer financial feature representations including technical indicators, volatility measures, macroeconomic variables, and market sentiment information. Such expanded feature spaces may improve the learning capability of hybrid quantum–classical architectures. Moreover, future work will compare the suggested hybrid framework against more robust current classical baselines, such as LSTM, GRU and Transformer-based sequence models and gradient-boosting models such as XGBoost, LightGBM and CatBoost, and will also factor repeat experiment runs with standard deviation, confidence intervals and formal significance test to statistically confirm the reported differences in comparative performance. The conclusions are drawn based on chronological forecasting evaluation, controlled comparison among models, and detailed performance analysis across multiple BSE indices.
